# Protocol of a test of hearing health education programs for farm and rural youth

**DOI:** 10.1186/s12889-015-2393-y

**Published:** 2015-10-16

**Authors:** Marjorie C. McCullagh, Tanima Banerjee, James Yang

**Affiliations:** University of Michigan School of Nursing, 400 N. Ingalls St., Ann Arbor, MI 48109 USA

**Keywords:** Hearing loss prevention, Hearing conservation, Farmers, Randomized controlled trial

## Abstract

**Background:**

Farm and rural youth have frequent exposure to hazardous noise on the farm and recreationally, and have an increased prevalence of noise-induced hearing loss. There is a lack of programs to prepare this high-risk population to use hearing conservation strategies.

**Methods:**

The purpose of this project is to test innovative hearing health education programs delivered to a large target group and to determine the effectiveness and sustainability of these programs in promoting hearing health among farm and rural youth. Specifically, this project includes: a) an interactive face-to-face informational program alone, b) an interactive face-to-face informational program followed by an Internet-based booster, and c) a no-intervention control. Sites will include selected affiliates of a major farm youth safety education organization. Data will be collected at baseline, 3, and 12 months. A linear mixed model will be used to compare the effectiveness of the three interventions over time. Descriptive statistics will be used to compare program costs and sustainability ratings.

**Discussion:**

Outcomes of this project will provide knowledge necessary to implement quality and cost-effective services to farm and rural youth, a high-risk and underserved population, that can be implemented and sustained after the study is completed.

**Trial registration:**

Clinicaltrials.gov NCT02472821 Registered 09 Jun, 2015.

## Background

Noise-induced hearing loss is a highly prevalent, permanent, irreversible condition that disproportionately impacts the future quality of life of farm and rural youth, their families, and communities. An estimated 2 million children and adolescents younger than 20 years of age are exposed to farm hazards as farm residents, farm family workers, hired workers, children of migrant or seasonal workers, or farm visitors resided on farms in 2010 [1]. Farm operators experience frequent exposure to high noise and among the highest prevalence rates of hearing loss among all categories of workers [2]. Similarly, farm and rural children have frequent exposure to high farm noise; [3] farm youth are engaged in farm tasks from an early age [4, 5]. In addition to their farm noise exposure, farm youth are exposed to high recreational noise (e.g., firearms, ATVs, and personal listening devices). Farm youth have lower hearing ability than their urban peers [6, 7].

Noise-induced hearing loss (NIHL) is permanent, irreversible, and insidious. Noise-induced hearing loss is characterized by loss of hearing in higher frequencies. The condition is permanent and incurable, and typically progresses slowly and insidiously with continued exposure to high levels of noise. Most people are unaware that they are affected until it is already moderately severe [8].

NIHL has a negative impact on the quality of life of the affected individuals as well as their families and communities. It affects physical and emotional functioning, social life, and employment. In addition, NIHL results in heavy social and economic burdens on families and communities from all ethnic and socioeconomic groups. In addition, persons with NIHL usually live with a lifetime *of* tinnitus, and have increased safety risks due to difficulty hearing warning sounds [9]. Monetary costs for NIHL are high, and include workers’ compensation (for employees) and medical costs [10]. Importantly, hearing loss has also been associated with increased risk for injury among farmers [11].

The significant features of the proposed studies are listed as follows:

1. *The proposed study maximizes the opportunity for success by focusing on primary prevention of hearing loss among youth.* Because NIHL is permanent and irreversible, treatments are limited to hearing aids for sound amplification. Most users find hearing aids expensive, unlike their natural hearing, and particularly unsatisfactory when there is background noise or when trying to focus on one speaker when there are other competing sounds [12]. The proposed study aims to change hearing health behavior from a young age, before the onset of noise-induced hearing loss, and before these youth add to the already high public health burden of persons with hearing loss [13].
2. *The proposed study tests the effectiveness of new evidence-based programs to protect a high-risk and underserved population of rural and farm youth from NIHL.* Farmers are a unique population. Unlike workers in general industry, family-owned farms with fewer than 11 employees are not protected by OSHA or its Hearing Conservation Standard (i.e., noise level monitoring, hearing conservation program, audiometric testing, training, provision of hearing protection devices) [14, 15]. It is also legal for minors younger than age 12 to be employed on a farm [16].
3. *The proposed study addresses barriers to use of hearing conservation strategies specific to rural and farm youth.* In a recent focus group study, farm youth reported that they were universally exposed to hazardous noise on the farm and in recreational activities,that perceived barriers to use of hearing conservation strategies outweighed the perceived benefits, and they lacked an association between their use of hearing conservation strategies today and their hearing ability later in life (invincibility). Noise elimination is the most preferred method of prevention of NIHL. However, this approach is often not technically or economically feasible in the farm work environment [17].
4. *The proposed study tests the sustainability of programs to protect rural and farm youth from noise-induced hearing loss.* Programs can continue to deliver benefits when they are sustainable, i.e., able to maintain programming and its benefits over time.
5. *The proposed study addresses the strategic goals of relevant federal agencies to reduce* noise-induced hearing loss and accompanying tinnitus, including Healthy People 2020, [18] NIDCD, [19, 20] NIOSH, [21] the National Agriculture, Forestry, and Fishing Agenda, [21] OSHA, [22] the North American Guidelines for Children’s Agricultural Tasks, [23] and IOM [24].
6. *The proposed study addresses barriers to adoption of evidence-based interventions that have been shown to be efficacious and effective.* Small-scale hearing conservation programs for farm and non-farm youth have been successful in changing HPD use behavior over short time periods, and offer confidence in the potential for maximizing the impact on health by changing behavior before the onset of noise-induced hearing loss, [5, 25–29] but effectiveness of these programs is limited by lack of systems to deliver programs widely to at-risk youth, and at an effective frequency to maintain change over time [25, 28, 30].
7. *The proposed study takes advantage of existing infrastructure.* The study involves partnering with well-established and culturally relevant systems that serve the health and safety educational needs of farm and rural youth (Progressive Agriculture Foundation Safety Day and Dangerous Decibels virtual exhibit). PAF does not currently offer hearing health education, but this new partnership will serve to add hearing health education to the already existing system designed to deliver health and safety educational programming to 98,000 rural youth and adults annually.
8. *The proposed study tests the effectiveness of a booster intervention to effect the desired public health impact.* Previous RCT studies examining the use of boosters on adult hearing protector use [31] have had mixed results, demonstrating the need for further research exploring the effects of the powerful intervention in this behavioral context among youth.

### Theoretical framework

Schell and colleagues [32] have developed a model for measuring eight organizational and contextual domains which best ensure that program outcomes can be realized over time [https://sustaintool.org/assess]. The proposed study seeks to assess the sustainability of hearing health education programs (with and without Internet boosters) using Schell’s model.

**Fig. 1:**
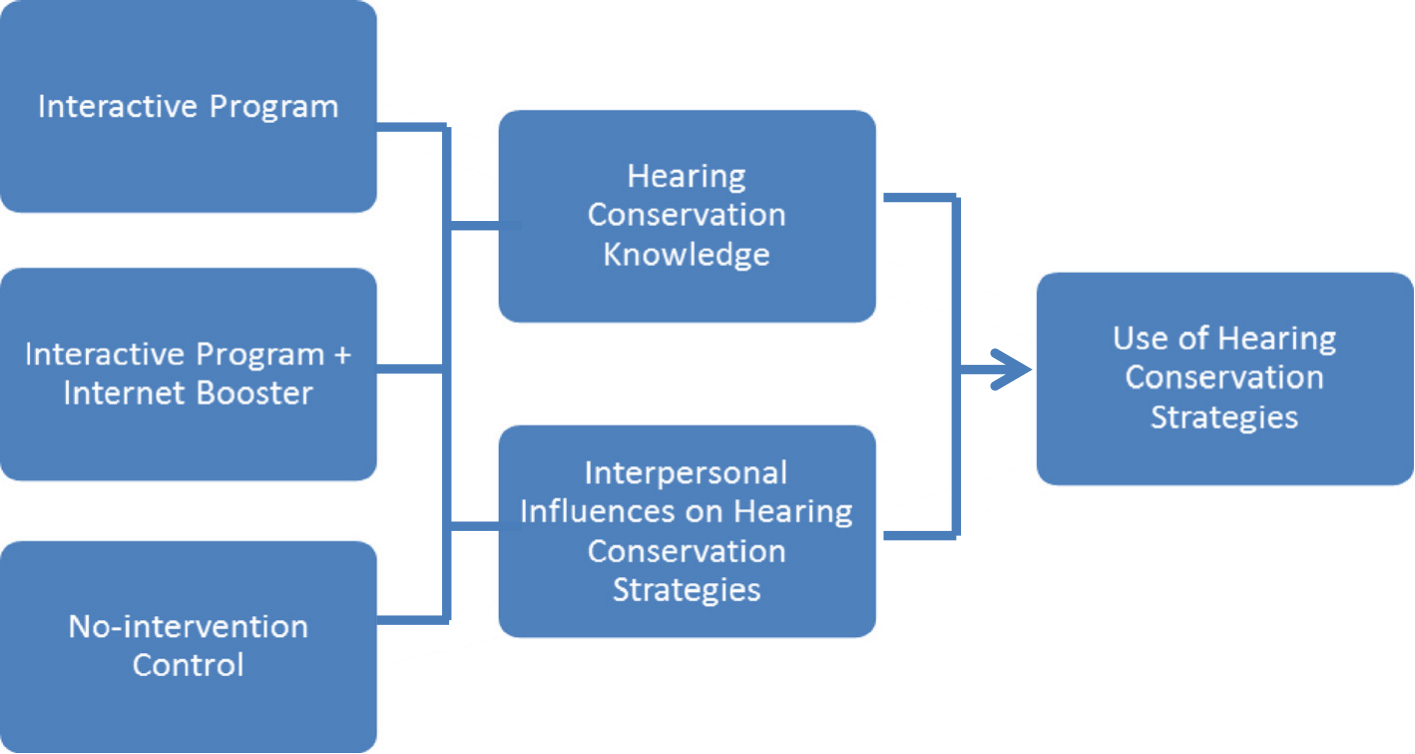
Intervention Effects on Use of Hearing Conservation Strategies

In addition, the intervention will be guided by the Model of Effects on Predictors of Intent to Use Hearing Conservation Strategies Intervention (Fig. 1).

Although hearing conservation programs for farm and non-farm youth have been successful in changing hearing protector use, [5, 25, 27–30] effectiveness of programs is limited by lack of reach to at-risk youth, and effective doses [25, 28, 30]. The proposed study uses an established educational system (Safety Day) to deliver hearing health education to youth, and will determine the most effective and sustainable approach to hearing health education among farm and rural youth.

This project will result in identification of the effectiveness, costs, and sustainability estimates associated with each approach to hearing health education among farm and rural youth. Cost-effective and sustainable hearing health education programs are expected to have the greatest impact on reducing rates of noise-induced hearing loss, tinnitus, and other negative effects of high noise exposure, and improving quality of life in this high-risk and underserved group.

This study maximizes opportunity for a sustainable impact on public health by building on a well-established educational program, Safety Day. Ongoing activities in the Safety Day (exclusive of this study) include recruiting, training, and administering consents; the study activities will incrementally increase activities in these areas, leading to a high level of efficiency.

The proposed study tests an under-tested intervention approach: the booster. Previous randomized clinical trial (RCT) studies examining the use of boosters on hearing protector use [31, 33, 34] have had ambiguous results, pointing to the need for further study of this promising approach.

The proposed study addresses long-standing problems with hearing health education programs which have not been addressed by previous effectiveness studies, i.e., access and program sustainability. The proposed study also uses a previously-established, tested, [28] free-to-use Internet program (Dangerous Decibels® virtual exhibit) not previously tested for use among farm and rural youth.

The proposed study uses a strong (RCT) scientific design. Information from this stronger study design will provide more generalizable evidence useful in the future development of hearing conservation programs and health policy, resulting in enhanced opportunity to impact the public’s health.

## Methods and design

A team of multidisciplinary members with diverse expertise has been formed to accomplish the study aims. Areas of expertise include: 1) development and testing of programs designed to increase use of hearing protection devices among farmers, 2) farm youth safety education, 3) analysis of hearing health education programs, 4) health program cost analysis, 5) assessment of public health program sustainability, and 6) Web hosting and user monitoring.

The purpose of this project is to compare the effectiveness and sustainability of new programs designed to increase hearing conservation practices (e.g., use of HPDs, turn it down, walk away) among farm and rural youth, thereby reducing noise-induced hearing loss, and involves a partnership with the Progressive Agriculture Foundation. Specifically, the study uses a cluster randomized-control design to compare two programs (with a control) for effectiveness and sustainability: a community-based interactive youth educational program alone (Group A); and a community-based interactive youth educational program followed by an Internet-based booster (Group B). Both programs will be delivered through the Progressive Agriculture Foundation (PAF) Safety Day program.

Safety Days are one-day, hands-on workshops that teach farm children safe farm practices. Each year, Safety Days reach more than 98,000 children and adults in over 400 sites across the United States and Canada. These fun, interactive, and hands-on learning experiences result in increased knowledge about safety, as well as positive change in safety behavior [35–38]. The intervention will also use the Dangerous Decibels® virtual exhibit (DDVE) an Internet-based educational program *targeted to youth* offered through Oregon Health Sciences University and partners. The DDVE will be used as *a* booster in the proposed study.

### Interventions

The study aims to test alternative hearing health education programs, and determine their effectiveness and sustainability for farm and rural youth.

Intervention A consists of the interactive face-to-face hearing conservation program developed by the project team. It consists of discovery-based, developmentally appropriate activities designed to teach common farm and recreational sources of hazardous noise, effects of hazardous noise on ear structure and function, and age-appropriate ways to protect oneself, e.g., walk away, turn it down, and wear protection. The 20-minute lesson includes student involvement in demonstrations and visuals, demonstration and supervised practice in insertion of a child-safe hearing protection device, provision of a sample hearing protector, and elements relevant to the rural and farm community (e.g., common farm noise sources). In a preliminary test with 64 fourth grade students, comparison of pre- and post-test results showed changes of scores in the desired direction, and pre-post-test differences were statistically significant*.* In addition, results of the pilot test suggested that overall, the pilot program was acceptable to school officials and students, instruments were reliable and valid, and the brief educational program is feasible.

Training in use of the face-to-face hearing health lesson plan will be provided to selected local safety program coordinators through face-to-face and virtual train-the-trainer sessions conducted by study personnel, and a written lesson plan; the Study Coordinator will also reach out to instructors by offering individualized training and consultation. Coordinators will prepare instructors, include the lesson in their local program, and complete surveys. The hearing health lesson includes use of easily accessible teaching materials, e.g., paper-based visual aids, locally-sourced noisy equipment (e.g. electric leaf blower), pipe cleaners, and a sound meter.

Intervention B consists of an Internet-based educational booster. The Internet-based booster reinforces Intervention A content through use of the Dangerous Decibels® virtual exhibit (DDVE), an existing online educational program consisting of eight activities (www.dangerousdecibels.org/exhibit/virtual-exhibit/). The booster is developmentally appropriate, focuses on sources of sounds, consequences of noise exposure, and prevention of hearing loss. In a study testing the effectiveness of the DDVE site when used alone, students showed improvement in knowledge immediately after the intervention, although improvements were not sustained after 3 months. The Internet-based booster is a user-driven activity where students explore a variety of learning activities at their own pace. Highly interactive game-based activities include rating noise sources (e.g., lawn mower) as safe or dangerous, determining the best course of action (i.e., turn it down, walk away, wear hearing protection) in a variety of noisy situations, and choosing a course of action when confronted with social non-support of use of hearing protection. A computer (with Macromedia Flash® and Shockwave®) and Internet connection (e.g., at home, school, or public library) is required for participation.

**Fig. 2:**
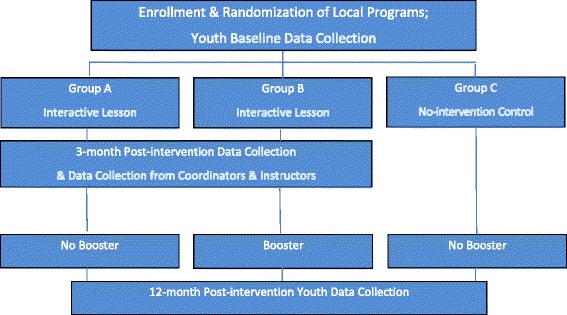
Study Design

The study design includes three arms: an interactive a face-to-face Safety Day lesson alone (Group A), a face-to-face interactive lesson followed by an Internet-based booster (Group B), and a no-invention control (Group C, Fig. 2). The Internet booster will be delivered to eligible participants following completion of the three month survey.

#### Setting and procedures

The study will take place through selected sites of a pre-existing system of community-based safety programs for farm and rural youth (Progressive Agriculture Safety Day). Local Safety Day programs will serve as clusters.

#### Subject sample

Farm and rural youth, as well as Safety Day instructors and coordinators will be sampled. Inclusion criteria for youth participants include enrollment in grade 4, parental consent, English speaking, and attending a Safety Day event included in the cluster sampling. Inclusion criteria for coordinators include demonstrated ability to successfully coordinate a Safety Day program, interest in the study, opportunity to implement the lesson in the designated study period, and willingness to comply with study procedures. Site selection will be aimed at including a diverse study sample, from the perspective of race, ethnicity, and geography. Inclusion criteria for instructors include accepting a hearing health teaching assignment at a Safety Day event included in the cluster sampling.

#### Recruitment

The study will use pre-existing Safety Day protocols for recruitment and consenting participants, with some modifications. Local Safety Day programs from diverse areas across the US will be offered the opportunity to enroll by a member of the study team. Recruitment of Safety Day instructors will be done in collaboration with the study team, who will collaboratively develop instructor recruitment strategies. Coordinators (and delegates) will recruit youth to participate.

#### Retention

With parental consent, youth will select their preferred communication media (telephone, postal, email), which will be used to communicate study activity reminders and surveys. The study coordinator will distribute reminders, and 3- and 12-month follow-up surveys by email, postal mail, or phone. Coordinators will access youth for 12 month follow-up as they return for Safety Day participation in the subsequent yearly meeting. The study team will support the PAF Safety Day trainers, coordinators, and instructors through site visits and frequent email and telephone contacts. Participating youth will be recognized with a $5 cash incentive for completing up to three (depending on study arm) surveys. Coordinators will be awarded checks on submission of study materials (surveys) as tokens of appreciation for their contribution to accomplishment of study goals (e.g., completion of training, coordination, & teaching; responding to survey requests).

#### Randomization

To reduce contamination of intervention effects across participants, randomization will occur at the level of local Safety Day programs. Participating Safety Day programs will then be randomly assigned to one of the three study conditions so that individuals within the same site receive the same intervention.

#### Process and outcome measures

Program process and outcomes will be measured by surveys completed by students, instructors, and coordinators. Instruments are described below, and instrument means, standard deviations, and alpha coefficients are displayed in Table 1.

**Table 1:** Instrument means, standard deviations, and alpha coefficients (n = 110)

Instrument Name	Number of items	Range of scores	Mean (SD)	Cronbach’s alpha
Knowledge	12	0.08-0.92	0.69 (0.18)	.71
Interpersonal Influences	1	1-4	2.45(1.17)	n/a
Use of HPDs & Strategies	6	0-1	0.67(0.27)	.69

#### Intervention effectiveness evaluation

After obtaining parental informed consent and soliciting the assent of child participants, pre- and post-intervention data will be collected from youths. Pre-test data will be collected via pencil-and-paper survey forms prior to the delivery of content. Three- and 12-month post-test data will be collected via the youth’s preferred communication medium following the booster intervention. Instrument means, standard deviations, and alpha coefficients are summarized in Table 2.

**Table 2:** Timetable of project activities and benchmarks for success

Activity	Year 1 months						Year 2 months						Year 3 months						Year 4 months		
	1/2	3/4	5/6	7/8	9/10	11/12	13/14	15/16	17/18	19/20	21/22	23/24	25/26	27/28	29/30	31/32	33/34	35/36	37/38	39/40	41/42
Recruit, enroll & randomize 36 sites	X	X	X	X																	
Train coordinators (Groups A,B); & distribute materials (Groups A,B,C)		X	X	X	X	X															
Recruit 24 instructors; enroll youth; collect baseline data; deliver interactive program (Groups A,B)					X	X	X	X													
Distribute, collect 3-month surveys (Groups A,B)					X	X	X	X	X	X											
Distribute booster reminders (Group B)							X	X	X	X	X	X									
Collect 12-month surveys; distribute incentives (Groups A,B,C)											X	X	X	X	X	X					
Complete sustainability assessment														X							
Analyze data															X	X	X				
Submit progress reports						X						X						X			X
Disseminate & submit manuscripts																			X	X	X

#### Use of hearing conservation strategies

The 3-item, multiple-choice instrument measures youths’ use of hearing conservation strategies (i.e., turn it down, walk away, use protection) during the previous 3-months. The instrument has a reading grade level of 6.9. A sample item from this instrument is, “During the past 3 months, how often did you wear earplugs or ear muffs when you were around loud sound?”

#### Intent to use hearing conservation strategies

The 6-item, multiple-choice instrument measuring youths’ intent to use hearing conservation strategies [28]. The instrument has a reading grade level of 5.6. A sample item from this instrument is, “*If earplugs were around when I needed them, I would use them*.”

#### Knowledge of hearing health

The 12-item, multiple-choice instrument to measure youths’ knowledge of noise, its effects on hearing, and hearing conservation strategies was adopted from items used by Martin et al. to measure this concept among elementary students [28]. A sample item from this scale is, “Which sounds can be loud enough to damage your hearing?”

#### Interpersonal factors influencing use of hearing conservation strategies

The 1-item, multiple-choice instrument to measure interpersonal factors influencing use of hearing conservation strategies among youth was adopted from a set of items used by Martin et al [28]. The item queries, “Wearing earplugs around your friends (if no one else is wearing them) would be (*very* to *not at all embarrassing*).” The instrument has a reading grade level somewhat higher (11.3) than the other instruments, due to use of the parenthetical phrase “*if no one else is wearing them*” and the multi-syllabic word, *embarrassing.* However, in previous use and pretesting, 4th grade students responded to this item without difficulty.

#### Process evaluation

Measurement of program process will include program fidelity, dose delivered, dose received, reach, recruitment, and context using instruments designed by the interdisciplinary study team for this purpose. The process evaluation plan was developed with consideration to system resources and program characteristics and context. Sample measurement items for the interactive Safety Day program follow. Fidelity will be measured by research team review of lesson audiorecordings for key points. Group C (control) site instructors will respond to a survey querying the inclusion of hearing conservation messages within the context of their sessions not specific to this topic (e.g., tractor safety). Dose of Intervention 1 will be measured using an instructor survey item, “How many minutes were spent on the lesson with each group of learners?” *Dose of intervention 2* will be measured as youth log into the DDVE site where they will enter identifying information which will allow for their time on the DDVE Web site to be tracked. Consistent with standard dose measurement methods, inactive time on site will result in stopping the time-on-site clock. The study team will monitor local Safety Day coordinators and instructors using phone calls and email messages timed to coincide with program planning, delivery, and follow-up to support delivery of the intervention as planned, and to accomplish submission of post-intervention surveys. *Reach* will be measured by counting the number of Safety Day youth (by site) who received the hearing health lesson. *Recruitment* will be measured using a coordinator survey item, “List and describe the youth recruitment procedures used.” *Context* includes the organizational factors, barriers, and facilitators to program delivery. Context will be measured using coordinator and instructor survey items, “What were the barriers to implementing the lesson?” and,”What were the facilitators to implementing the lesson?”

#### Demographics

This 3-item instrument measures youths’ sex, age, and race/ethnicity.

#### Cost

The cost of implementing the program will be based on data associated with program personnel, instructional materials, and travel incurred with the educational process.

- Personnel (coordinators, instructors, youth): We will estimate hearing education program personnel costs beyond those already incurred through previously established Safety Day programs, i.e., youth, coordinator, and instructor time.
- Instructional materials: We will value all materials used to implement the lesson (e.g., sound meters, printed supplies), including lesson development costs and youth time on public-access or home-based computers.
- Travel: We will estimate travel costs incurred by coordinators, instructors, and youth to participate in pre-program training and the local Safety Day program.

#### Sustainability

Schell’s [32] tool will be used to assess sustainability of the hearing health education program. The tool consists of 8 domains, each with five items. For example, the program adaptation domain includes an item, “The program periodically reviews the evidence base.” Following Safety Day intervention delivery, program coordinators will respond to an electronic survey, rating each factor for their own site on a 7-point Likert scale anchored with response options, *to a little or no extent* and *to a great extent.*

### Power and sample size justification

The sample size, in particular the number of sites, was determined by power analysis for a mixed model design using Optimal Design software [39]. The number of sites was selected to provide 80 % power to detect an effect of either intervention with effect size *d* of .48 compared to the effect of the control treatment. This is the effect size on intention to use hearing conservation strategies we found in our preliminary study and is just under what Cohen defined as a medium sized effect. Other necessary inputs into Optimal Design were ranges of the average number of students per site and estimates of the intraclass correlation coefficient (ICC), a measure of the tendency of individuals in the same site to have similar (correlated) responses. To determine the number of sites needed, we examined numbers of site ranging from 20 to 80, and a value of the ICC of .10 (which is typical of its value in health education intervention research [40]. We also adjusted for the possibility of 20 % missing posttest data (which we did by reducing the number of individuals per site by that percentage: thus actually testing the number of individuals per site from 16 to 64). These last adjustments increased the number of clusters we needed to include. The result of the power analysis was 80 % power to detect the target effect size with alpha of .05 two tailed if we included 36 sites with an average 16 individuals per site. Thus we have designed the study to include 36 sites (divided equally among the 3 treatments). Assuming each site includes more than 16 individuals, we will have more than 80 % power. Target youth enrollment of 576 is based on these calculations and US Agriculture Census data [41]. Enrollment numbers also include coordinators (n = 36) and instructors (n = 24).

### Statistical analysis

Survey data will be stored on the password-protected, user-authenticated encrypted computer hard drive. Before conducting analyses to address the aims, preliminary analyses will be conducted to evaluate the quality of data and to fix illegal values.

*Aim 1 will compare effectiveness of two interventions* (community-based interactive youth educational program and community-based interactive youth educational program plus Internet booster) *to the no-intervention control treatment.* A secondary component of Aim 1 is to compare two interactive hearing interventions on youth.

The primary measure to assess effectiveness is the 6 item scale of intention to use hearing conservation strategies (including turning down the volume, moving away from the noise, and using hearing protection devices such as ear plugs). The knowledge scale and the scale of interpersonal influences will also be examined. All individuals will be asked to assess these measures before intervention (baseline), and at 3 months and 12 months after the intervention. Since the cluster randomized design will be used to assign interventions and repeated measurements will be collected from the same individuals, linear mixed effects model will be used to investigate the effect of the interventions. The outcome variable is the scale of intention to use hearing conservation strategies. The important/main fixed effect is a categorical variable identifying the type of interventions. In addition, demographics variables will be included in the mixed model as fixed effects to increase the precision of estimates. The random effect in the model is the *site* variable. More precisely, we will use random intercept model for the *site* variable. We assume the site variable follows a normal distribution with mean zero and is independent to the error terms in the mixed model. The hypothesis is that there will be a significant effect of hearing intervention on the use of hearing conservation strategies and the intervention group will have higher use of hearing conservation strategies than the control group. If the effect of intervention is significant, follow up descriptive statistics on mean intent by time by treatment group will allow us to describe the effects, for example to determine whether each treatment led to more improvement in intent to use than the control condition and how big those effects were. Further tests within this analysis will compare the effects of the two interactive hearing interventions on youth.

#### Aim 2 will compare program costs

Costs in dollars (e.g., material, travel) will be summarized using mean and standard deviation for each site. Costs in time from volunteers and students will be summarized using mean for each site. At site level, the difference in cost between three intervention groups are compared using one-way analysis of variance. If the effectiveness of intervention is significant, the costs to effective ratio will be calculated to quantify incremental cost of effectiveness.

#### Aim 3 will compare sustainability of the programs

Safety Day coordinators (Groups A & B), program leaders, and research team members will complete the 40 item questionnaire rating 8 domains of sustainability. Two sample *t*-test or chi-squared test will be used to compare the difference between groups A and B. These testing results will help identify strengths and weaknesses of the Safety Day program (and the hearing conservation strategies within them) to pinpoint where efforts can be made to enhance the sustainability of the programs. Subsequently, a panel of Safety Day leaders and study team members will meet via teleconference to compare sustainability assessments of the two programs, and implications for program development.

### Study challenges and proposed solutions

#### Child participation

The study procedures include measures to protect children from coercion. Parental consent will be obtained prior to data collection; children who are not enrolled in the study will also receive the Safety Day intervention, but no data will be collected from them. Safety Day personnel are well-known local community members who are invested in the health and safety of youth in their own community, and often have a history of volunteering with the program for many years.

#### Self-reported measures

The study effectiveness measures are based on participant self-report, which is vulnerable to social desirability bias. However, in a nationwide sample of farmers participating in a test of hearing conservation interventions, participants scored low on a standard measure of social desirability reporting.^81^ In other reports, self-report and observations were highly correlated [42, 43].

#### Response rate, retention, and missing data

Successful recruitment and retention strategies used in previous longitudinal studies by McCullagh will be used in this project to maximize enrollment and reduce attrition; these strategies have resulted in consistently high study recruitment (e.g., 115 % of target) and retention (e.g., 90 % at 6 months, 91 % at 12 months) across studies, and include attention to communication with participants, high regard for participant autonomy, careful recordkeeping of participant progress through the study, and fastidious attention to follow-up. For missing data that does occur, we will use multiple imputation to impute missing values while providing a theoretically appropriate indication of uncertainty assuming the data are missing at random [44]. The same multiple imputation model based on baseline data will be used in all three treatment conditions.

#### Language and characteristics of farm and rural youth

The PAF Safety Day program is delivered in the US exclusively in English, and primarily to residential (non-migrant) youth. This is a significant limitation of the study, and an area for future program development.

##### Dissemination

This translational study design facilitates the transition of the program from research study to full-scale implementation within the Safety Day program, should study results demonstrate satisfactory effectiveness, increasing access to hearing conservation services to an usually large audience in a very short period of time. The potential exists for adding additional learning activities for use in repeating the lesson in subsequent years to the same youth. Also, opportunities for distributing the program through other venues will be explored, e.g., NIDCD website, selected teacher associations (e.g., National Science Teachers Association), and selected Web-based lesson planning resources used by science, health, and elementary teachers, as the program includes many components of state *and national (*e.g.*, Core Curriculum)* educational benchmarks for science, physics, math, and health education.

## Discussion

Farm youth are exposed to farm noise hazards as farm residents, farm family workers, hired workers, children of migrant or seasonal workers, or farm visitors, and have an increased prevalence of noise-induced hearing loss. Although previous tests of programs to promote hearing conservation among farm youth have demonstrated increases in hearing protector use or intention, their impact has been limited by program scope, cost, and sustainability.

This proposed study capitalizes on partnerships with existing infrastructures (i.e., PAF, DDVE). These partnerships will serve to incorporate hearing health education into already existing systems designed to deliver health and safety educational programming to rural students. The interventions are aimed broadly at situation-appropriate hearing conservation strategies (e.g., turn it down, walk away), and not limited to use of hearing protection devices. The proposed interventions are developmentally appropriate, use a highly interactive and discovery learning approach, were successfully pilot tested, and are ready for RCT.

This RCT test of interventions is designed to determine the most cost effective and sustainable approach to increasing use of hearing conservation strategies among farm and rural youth. Further, given effectiveness of the interventions, a system is already in place to disseminate programs to reach a larger farm youth audience. Results of this study will inform future intervention studies, interventions aimed at farm youth, and interventions to increase use of hearing conservation strategies, as well as offer a base for developing programs for non-English speaking children.

A strong team of researchers and the largest farm youth health and safety education program in the US have formed a partnership in an effort to address this important health problem in the interest of reducing hearing loss and tinnitus, and improving the quality of life of this high risk and underserved group.

### Ethics, consent and permissions

The study protocol was reviewed by the University of Michigan Health Sciences and Behavioral Sciences Institutional Review Board (HUM00077214) who determined that the proposed research conforms to applicable guidelines, State and federal regulations, and the University of Michigan’s Federalwide Assurance (FWA) with the Department of Health and Human Services (HHS). Parental consent will be obtained prior to data collection from youth; adult participants will be required to provide their own consent.
